# Integrated microRNA, mRNA, and protein expression profiling reveals microRNA regulatory networks in rat kidney treated with a carcinogenic dose of aristolochic acid

**DOI:** 10.1186/s12864-015-1516-2

**Published:** 2015-05-08

**Authors:** Zhiguang Li, Taichun Qin, Kejian Wang, Michael Hackenberg, Jian Yan, Yuan Gao, Li-Rong Yu, Leming Shi, Zhenqiang Su, Tao Chen

**Affiliations:** Institute of Cancer Stem Cell, Second Affiliated Hospital, Cancer Center, Dalian Medical University, Dalian, 116044 China; Division of Genetic and Molecular Toxicology, National Center for Toxicological Research, Food and Drug Administration, 3900 NCTR Road, Jefferson, AR 72079 USA; Division of Systems Biology, National Center for Toxicological Research, Food and Drug Administration, 3900 NCTR Road, Jefferson, AR 72079 USA; Genetics Department, Facultad de Ciencias, Universidad de Granada, Granada, 18071 Spain; Current address: School of Pharmacy, Fudan University, Shanghai, 201203 China

**Keywords:** Aristolochic acid, Carcinogenesis, Kidney tumor, microRNA, Proteomics, Deep-Sequencing, RNA array, Target prediction, Rat

## Abstract

**Background:**

Aristolochic Acid (AA), a natural component of Aristolochia plants that is found in a variety of herbal remedies and health supplements, is classified as a Group 1 carcinogen by the International Agency for Research on Cancer. Given that microRNAs (miRNAs) are involved in cancer initiation and progression and their role remains unknown in AA-induced carcinogenesis, we examined genome-wide AA-induced dysregulation of miRNAs as well as the regulation of miRNAs on their target gene expression in rat kidney.

**Results:**

We treated rats with 10 mg/kg AA and vehicle control for 12 weeks and eight kidney samples (4 for the treatment and 4 for the control) were used for examining miRNA and mRNA expression by deep sequencing, and protein expression by proteomics. AA treatment resulted in significant differential expression of miRNAs, mRNAs and proteins as measured by both principal component analysis (PCA) and hierarchical clustering analysis (HCA). Specially, 63 miRNAs (adjusted p value < 0.05 and fold change > 1.5), 6,794 mRNAs (adjusted p value < 0.05 and fold change > 2.0), and 800 proteins (fold change > 2.0) were significantly altered by AA treatment. The expression of 6 selected miRNAs was validated by quantitative real-time PCR analysis. Ingenuity Pathways Analysis (IPA) showed that cancer is the top network and disease associated with those dysregulated miRNAs. To further investigate the influence of miRNAs on kidney mRNA and protein expression, we combined proteomic and transcriptomic data in conjunction with miRNA target selection as confirmed and reported in miRTarBase. In addition to translational repression and transcriptional destabilization, we also found that miRNAs and their target genes were expressed in the same direction at levels of transcription (169) or translation (227). Furthermore, we identified that up-regulation of 13 oncogenic miRNAs was associated with translational activation of 45 out of 54 cancer-related targets.

**Conclusions:**

Our findings suggest that dysregulated miRNA expression plays an important role in AA-induced carcinogenesis in rat kidney, and that the integrated approach of multiple profiling provides a new insight into a post-transcriptional regulation of miRNAs on their target repression and activation in a genome-wide scale.

**Electronic supplementary material:**

The online version of this article (doi:10.1186/s12864-015-1516-2) contains supplementary material, which is available to authorized users.

## Background

Aristolochic acid (AA) is found in plants of the genus *Aristolochia* and *Asarum* [1]. Use of dietary supplements and other botanical products containing AA has caused severe nephrotoxicity and consequent renal replacement therapy [2,3]. Animal studies show that AA results in renal failure in rodents and induces tumors in the kidney and other tissues of rabbits, rats and mice [4,5]. AA is among the most potent 2% of the carcinogens in the Carcinogenic Potency and Genotoxicity Databases. As a result, the U.S. Food and Drug Administration (FDA) issued a Consumer Advisory in 2001 warning consumers against using dietary supplements and other botanical products containing AA, and the FDA also requested a recall of these products and published a list of botanical products that contained AA. However, products containing AA have not been banned in the US or many other countries. AA-induced carcinogenesis has been attributed to the mutagenicity and DNA adducts formed in the kidney and other tissues of AA nephropathy patients. On the other hand, AA induced similar DNA adduct formation in both the kidney and liver of mice, but tumors preferentially occurred in kidney [6]. This suggests that in addition to the genetic alterations induced by AA, alternative mechanisms such as epigenetic remodeling and miRNA (miRNA) modulation might also play an important role in AA-induced cancers.

miRNAs represent a class of non-coding small RNA (~22 nt) that are ubiquitously present in different kinds of organisms from *C.elegans* to mammals [7,8]. miRNAs are involved in the post-transcriptional regulation of gene expression via binding to the 3’ UTR region of target mRNAs, resulting in mRNA degradation or translation inhibition [8]. Each miRNA usually targets multiple, even hundreds of mRNAs [9]. It is believed that one third of human genes are subject to miRNA control. miRNAs regulate a variety of developmental and physiological processes, including control of leaf and flower development in plants [10] and neuronal patterning in nematodes [11]. Recent studies indicate that miRNAs are involved in the regulation of pathways that are associated with the initiation and progression of many types of tumors [12-14]. miRNA expression was found to accurately identify the tissue origin of cancers, including distal metastatic colonies of unknown primary origin [15]. miRNAs affect tumor metastasis, such as the lung and bone metastasis of human breast cancer [16]. Moreover, miRNAs are associated with chemical carcinogenesis and regulate gene expression that is important in every stage [17].

Deep sequencing, also known as next generation sequencing, has undergone tremendous acceptance in the past few years. By sequencing DNA or RNA in a massively parallel fashion, deep sequencing technologies dramatically reduce both the cost-per-base and time required to decode an entire genome or transcriptome [18-20], making sequencing a cost-effective option for many experimental approaches. Moreover, it has very low, if any, background signal, and does not have an upper limit for quantification, resulting in a large dynamic range of expression levels over which transcripts can be detected [21]. Toxicoproteomics is a new discipline that applies proteomics concepts and approaches to toxicological studies. It can elucidate pathological responses to a specific toxicant at the protein molecule level, including qualitative and quantitative measurements of protein expression, protein modifications, and protein-protein/toxicant interactions [22].

A primary research objective of miRNA biology is to understand the post-transcriptional regulation of the target genes and the effect on biological functions. However, due to the different mechanisms of miRNA regulation, the targets can be translationally repressed with a concordant decrease in mRNA abundance or without significant mRNA degradation, or have decreased mRNA abundance with little protein change [23-25]. Moreover, miRNAs can switch from repression to activation depending on different stages of the cell cycle [26]. Therefore, in order to understand miRNA regulation in AA-induced kidney tumorigenesis, we integrated deep sequencing data for global miRNA and mRNA expression with proteomics data for global protein expression in conjunction with target identification of dysregulated miRNAs from the miRTarBase [27,28]. Compared with using miRNA target prediction tools such as miRanda and TargetScan [29,30], we selected those targets that were published and validated to study their miRNA transcriptional and translational regulations [31,32], thus providing a novel approach to enhance our understanding of the biological role of miRNA in AA-induced carcinogenesis in rat kidney. The details of the proteomics data from rats treated with AA were reported separately [33]. This work will contribute to our understanding of the biological processes driving AA-induced carcinogenesis at the miRNA, mRNA and protein levels as well as representing a generalizable strategy that can be extended to study the functional role of miRNAs and their regulation of target expression in organs or tissues of interest.

## Results

### AA treatment resulted in functionally differential miRNA expressions in rat kidney

**Figure 1:**
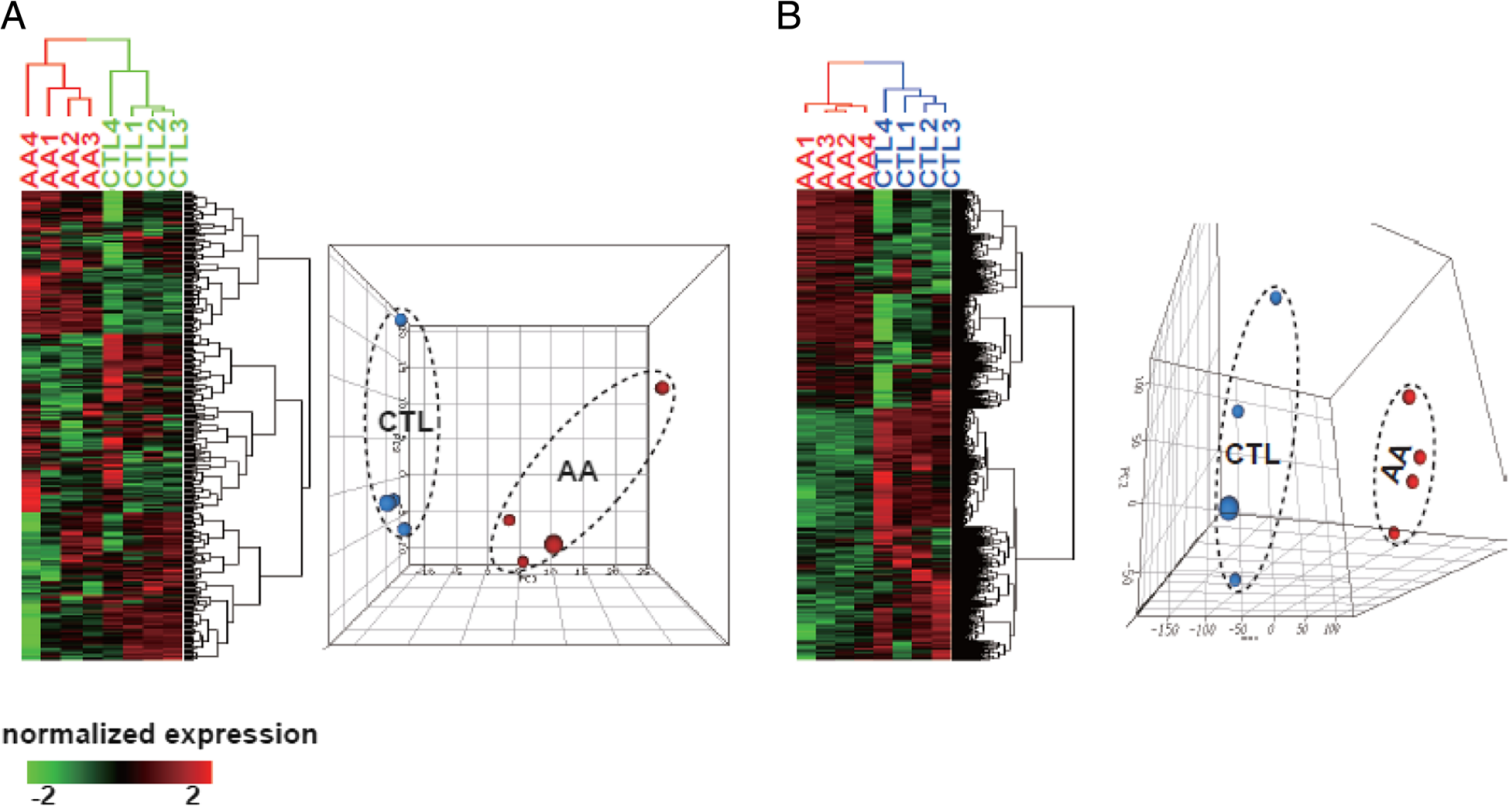
Identification of differentially expressed miRNA and mRNA in kidney in rat treated with AA. **A**. Classification of samples according to expression intensity of the 417 detectable miRNAs. Hierarchical clustering analysis separates the 8 samples into two groups that are consistent with control and the treatment groups. Principal component analysis groups samples into the AA- treated and control clusters. **B**. Classification of 8 samples according to expression level of the 23939 detectable mRNAs. Hierarchical clustering analysis separates the 8 samples into two groups that are consistent with the control and treatment groups. Principal component analysis groups samples into the AA- treated and control clusters.

Dysregulated miRNA expression has been linked to cancer in experimental animal models and patients [34]. To investigate the effects of AA exposure on miRNA expression under conditions that resulted in kidney tumors, we treated 4 rats for 12 weeks with AA along with 4 control rats treated with vehicle, following a carcinogenetic protocol that has been demonstrated to result in kidney tumors [35]. All the rats were sacrificed one day after the last treatment and the kidneys were removed for miRNA deep sequencing analysis. The short reads were mapped to Sanger miRBase (miR 16) [36]. There were 417 detectable miRNAs in at least one of the 8 samples. First, classification analyses were conducted to explore global alteration of miRNAs by the AA treatment. Hierarchical clustering analysis (HCA) was performed to globally view the groups among the 8 samples in terms of the expression profile of all the detectable miRNAs. These samples are divided into two branches, one comprises 4 AA-treated samples and the other contains the 4 control samples. This indicates that miRNAs have similar and consistent expression patterns within each group, but are distinct from each other, demonstrating the profound impact of AA treatment on miRNA expression in rat kidney (Figure 1A). The same phenomenon was revealed by the principal component analysis (PCA) analysis, further supporting that miRNA expression is profoundly changed by AA treatment at the genome scale.

Next, we investigated which miRNAs were significantly dysregulated by AA treatments. All the 417 detectable miRNAs were used to find differentially expressed miRNAs (DEM). Read count normalization, statistical analysis and p value adjustment were performed using the DEseq methods. Sixty-three miRNAs showed significant changes in their expression level by AA treatment using a cutoff with adjusted p values of < 0.05 and fold change of > 1.5 (Additional file 1: Table S1). More up-regulated miRNAs (n = 43) were observed than down-regulated miRNAs (n = 20).rno-miR-881, rno-miR-880, miR-741-3p, miR-511*, miR-187, miR-449a, as well as 6 members of miR-34 family, miR-34a, miR-34a*, miR-34b, miR-34b*, miR-34c, and miR-34c*, showed over 10-fold up-regulation. In contrast, only one miRNA, miR-383, showed more than 10-fold down-regulation. Moreover, the oncogenic miRNA, mir-21 was increased 3.8 fold, whereas the tumor suppressor miRNAs, Let-7e, mir-135a, and mir-375 were decreased by 2.9, 2.7 and 5.9 fold, respectively. In order to validate the changes in miRNA expression detected by a deep-sequencing, we conducted quantitative real-time PCR analysis to examine expression levels of six miRNAs including rno-miR-378, rno-miR-182, rno-miR-21, rno-miR-34a, rno-miR-34b, and rno-miR34c. The results from the real-time PCR analysis confirmed the sequencing data (Additional file 2: Figure S1).

Finally, 63 miRNA target proteins that were dysregulated in rat kidney were inputted into the Ingenuity database to explore the underlying biological and toxicological functions. The biological function analysis showed that cancer was the top functional category significantly related to these miRNAs, along with organismal injury and abnormalities, which also could result from AA treatment. In total, 31 of the 63 miRNAs were cancer-related (Table 1).

**Table 1:** **IPA analysis of 63 dysregulated miRNAs in rat kidney treated with AA**

**Top networks (score)**	**Top diseases and functions**
Cancer, Organismal Injury and Abnormalities (30)	Cancer
Cancer, Endocrine System Disorders (25)	Organismal Injury and Abnormalities
Cancer, Hematological Disease, Immunological Disease (21)	Reproductive System Disease
Drug Metabolism, Lipid Metabolism, Molecular Transport (8)	Endocrine System Disorders
	Gastrointestinal Disease

### Differentially expressed genes (DEGs) from mRNA sequencing data and their functions

miRNAs regulate the homeostatic level of their target mRNAs. To investigate whether mRNA expression was also changed by AA treatments, the 8 rat kidney samples were analyzed by mRNA deep sequencing analysis in parallel. The short reads were mapped to Ensembl transcripts (RGSC3.4), and a data processing procedure similar to that used for the miRNAs was applied to normalize the raw counts, and to calculate adjusted p values. There were 23939 mRNAs that had detectable expression in these 8 samples. As with miRNA expression in rat kidney, the expression patterns of mRNA were similar within the treatment or control group alone, but distinctly between the two groups in both HCA and PCA analysis (Figure 1B). A total of 6794 richness mRNAs were found to be differentially expressed based on a cutoff of p < 0.05 and fold change > 2, with 4051upregulated and 2743 down-regulated. Ingenuity Pathway analysis (IPA) of both up-regulated and down-regulated genes identified cancer as the top disease and biological function with 1197 up-regulated and 685 down-regulated molecules included (p = 1.48-22E-1.09E-06 and p = 4.95-14E-1.64-02E, respectively). The top canonical pathways included WNT/β-actin, mitochondrial dysfunction and valine degradation. (Additional file 3: Figure S2).

### An integrated multiple profiling reveals a comprehensive regulation of miRNAs on their target expression

In order to search for the targets of its differentiately regulated miRNAs and understand effects of the post-transcriptional regulation of miRNA on gene expression in AA-induced carcinogenesis, we combined proteomic and transcriptomic data to study expression of the miRNA targets that are validated and reported in miRTarBase (http://mirtarbase.mbc.nctu.edu.tw/). This database provides the most current and comprehensive information on experimentally validated miRNA-target interactions and data resources with published studies on the identification of miRNA targets, molecular networks of miRNA targets and systems biology. Specifically, it contains 5224 miRNA targets that are validated by one or more solid experimental methods including luciferase reporter assay, western blot, northern blot, and quantitative real-time analysis. If a gene is targeted by multiple miRNAs, the log2 transformed fold changes of these miRNAs were summed together as the fold changes of the miRNAs.

We initially conducted correlation analysis between the miRNAs vs. mRNAs, miRNAs vs. proteins, and mRNAs vs. proteins in rat kidney treated with AA in relation to the control. There was a very weak correlation between expression of mRNAs versus proteins (R = 0.02, P < 0.001), and miRNAs vs. proteins (R = 0.003, P <0.01), but there was no correlation between miRNA vs. mRNA expression (R = 0.002, P =0.85) (Additional file 4: Figure S3), indicating that regulation by miRNAs on their target genes can be attributed to multiple mechanisms.

**Figure 2:**
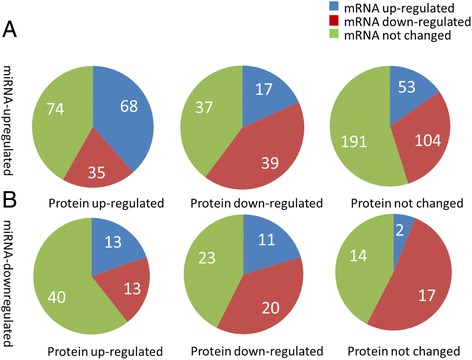
miRNAs regulated their target gene expression. **A**. genome-wide mRNA and protein expression associated with down-regulated miRNAs in rat kidney. We identified 63 dysregulated miRNAs and selected their targets that are validated and reported from miRTarBase and correlated with their mRNA and protein expression. **B**. Genome-wide mRNA and protein expression associated with up-regulated miRNAs in rat kidney.

Next, we divided our study into two groups including group A, the up-regulated miRNAs and group B, the down-regulated miRNAs, and used an adjusted p < 0.05 and fold changes of < -1.5 or > 1.5 as a cutoff, indicating the difference for all these three variables. According to the response of gene expression in group A, we separated our study into three subgroups: 1) mRNAs and proteins expressed in the same direction including up-regulated (68), down-regulated (39), not changed (191). 2) mRNAs and proteins expressed in the opposite direction including 35 down-regulated mRNAs vs. up-regulated proteins and 17 up-regulated mRNAs vs. down-regulated proteins, and 3) miRNA targeted gene translation indicated by protein up-regulated (74) or down-regulated (37) without a change in mRNA expression, or targeted transcription indicated by mRNA up-regulated (53) or down-regulated (104) without a change in protein expression (Figure 2A).

Similarly, for group B, we separated our study into three subgroups: 1) mRNAs and proteins expressed in the same direction including both up-regulated (13), down-regulated (20), and not changed (14); 2) mRNAs and proteins expressed in the opposite direction including 13 down-regulated mRNAs vs. up-regulated proteins and 11 up-regulated mRNAs vs down-regulated proteins, and 3) miRNA targeted translation indicated by protein up-regulated (40) or down-regulated (23) without a change in mRNA expression, or targeted transcription indicated by mRNA up-regulated (28) or down-regulated (17) without a change in protein expression (Figure 2B).

In summary, miRNAs exhibited a more complex regulation on their target expression, indicated by both repression and activation at levels of transcription and translation. Also, miRNAs affected gene translation without a change in mRNA abundance and vice versa. The correlation of expression between mRNAs and proteins was very weak, indicating that post-transcriptional regulation by miRNAs or other factors are involved in this regulation.

### miRNA-target interaction networks associated with cancer in rat kidney treated with AA

**Figure 3:**
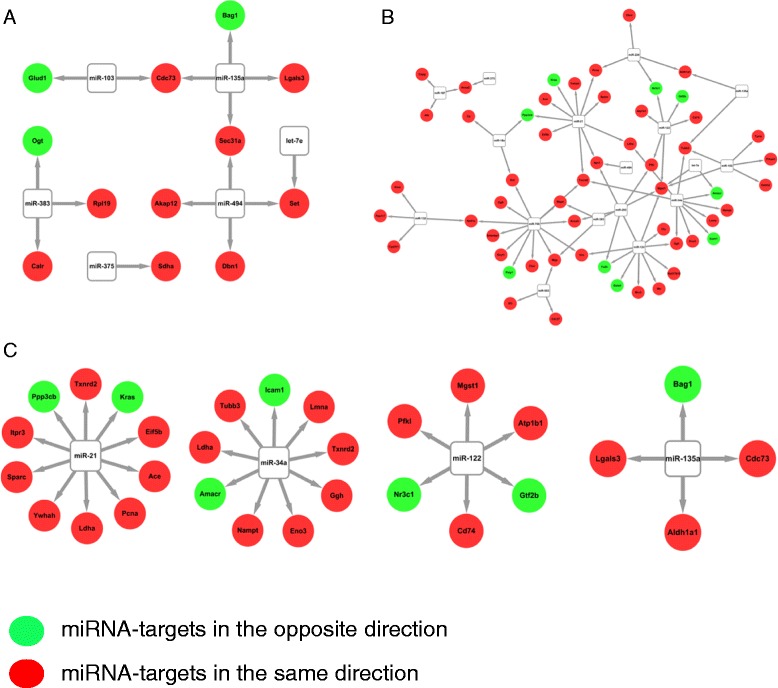
Interaction of miRNAs with their cancer-related protein targets. **(A)** miRNA targets were selected from IPA analysis and matched to miRNAs that were down-regulation. **(B)** miRNA targets matched to miRNAs that were up-regulated. miRNA targets – protein expression that has the same direction indicated by the red color and opposite direction indicated by the green color. **(C)** Highlighted miRNA-target interactions of miR-21, miR-34a, miR-122, and miR-135a.

We examined the interactions of miRNA with cancer specific targets that were expressed at the protein level and more related to their functional regulation. We used IPA analysis to pull out all the cancer-related miRNAs' targets from miRarBase and matched with 31 dysregulated miRNAs associated with cancer in rat kidney treated with AA. Therefore, we created miRNA regulation networks that link miRNAs and their targets to AA-induced carcinogenesis in rat kidney. For example, we identified 12 cancer-related targets associated with down-regulated miRNAs. Among these, 9 targets were down-regulated for protein expression and only 3 were up-regulated (Figure 3A). Also, 54 cancer related targets were associated with 13 up-regulated oncogenic miRNAs. Among these, 45 targets were up-regulated for protein expression, and only 9 were down-regulated (Figure 3B). In other words, miRNAs exhibited a bi-directional regulation of their targets and most cancer related target expression had the same direction as the miRNA expression, an unexpected finding.

We next selected several few specific oncogenic miRNAs and examined their targets' expression. For example, miR-21 was up-regulated in rat kidney. The expression of its target genes Ppp3cb and Kras were down-regulated, but eight target genes including YWHAH, TXNRD2, LDHA, ACE, EIF5B, SPARC, ITPRC, AND PCNA were up-regulated, and these targeted genes are related with several cancer signaling pathways including ERK/MAPK, PI3K/AKT, and 14-3-3 mediated signaling. miR-34a was up-regulated. The expression of its target genes LCAM1 AND AMACR were down-regulated, but 7 out of 9 miR-34a's targets were up-regulated including TUBBS, LDHA, NAMPT, ENO3, GGH, TXNRD2, and LMNA which are associated with mitochondrial dysfunction, DNA damage and repair, and apoptosis (Figure 3C).

## Discussion

The International Agency for Research on Cancer (IARC) has classified herbal remedies containing plant species of the genus *Aristolochia* as human carcinogens (IARC, 2002). In the present study, we profiled the expression of miRNA, mRNA and protein in the kidney of rats treated with a carcinogenetic dose of AA. We also employed an integrative methodology to study the correlations among miRNA, mRNA, and protein expression during AA-induced kidney carcinogenesis. We found that miRNA expression was significantly changed by AA treatment, with 63 altered miRNAs, accounting for 15% of 417 detectable miRNAs. We performed real-time PCR to validate the miRNA expression changes detected by deep-sequencing analysis. Moreover, in a genome-wide analysis, we found that altered miRNA expression had different effects on their target at the levels of both transcription and translation. We further determined that miRNA regulation is associated with both gene repression and activation, which could help us better understand miRNA biology in AA-induced rat kidney tumorigenesis.

miRNA dysregulation is recognized as a driving force in human cancer. Alteration of miRNA expression is commonly observed following exposure to different carcinogens [17,37,38], but the role of miRNAs in AA-induced carcinogenesis was not studied previously. Genetic changes are frequently observed in experimental animal models treated with AA. For example, we have previously shown that AA induces a high frequency of DNA mutations, with A:T → T:A transversion as the predominant mutation type [6]. In addition to the genetic changes induced by AA, we have found that AA treatment result in dysregulation of miRNAs and that half of them are associated with cancer according to IPA analysis. We selected up-regulation of oncogenic miRNAs such as mir-21 as well as down-regulation of the tumor suppressor miRNAs Let-7e, mir-135a, and mir-375 for our analysis. In patients, miR-135a inhibits cancer cell proliferation and exhibits the properties of a tumor suppressor in renal-cell carcinoma [39], while its target oncoprotein BAG-1 increases more than 4 fold as determined by proteomics. A recent study demonstrates that AA treatment altered a number of miRNAs in proximal tubular epithelial cells such as up-regulation of miR-192, miR-194, miR-450a, and miR-542. miR-192 mediated DNA damage response and recapitulated G2/M arrest via repression of murine double-minute 2, a negative regulator of P53 [40]. Similarly, we found that AA treatment increased expression of miR-450a and miR-542, and that AA induced a DNA damage response indicated by up-regulation of miR-34a. It was followed by activation of P53 and the binding P53 to the promoter of miR-34a resulted in mir-34 activation [41]. On the other hand, we did not see any consistency of 9 down-regulated miRNAs as identified in that study. This discrepancy could be explained by the fact that we used an in vivo model to study AA-induced carcinogenesis, which might be affected by many factors such as microenvironment, immune factors, and so on. All of these are not modeled in an in vitro study. It is likely that the carcinogenic effects of AA depend upon the balance between the oncogenic miRNAs and tumor-suppressor miRNAs. Thus, the alterations in these miRNAs might play an important role in promoting tumor development in rat kidney as well as inducing kidney toxicity, suggesting that identifying the miRNA targets involved in these processes are important.

We combined multiple levels of profiling to study miRNA target expression and identified those targets of dysregulated miRNAs that are validated and reported from miRTarBase. Currently, miRNA target identification is primarily based on computational predications that could result in a vast number of targets with errors, raising the problem of identifying functional targets for validation [42]. From our knowledge, this is the first time that this novel approach has been used to study the relationship among three levels of expression of miRNA, mRNA and protein on a genome-wide scale. Even though there might be some missing targets for miRNAs identified in this study in the miRTarBase, we have identified several categories of miRNA-target relationship that might represent general miRNA regulation patterns and the biological operating during AA-induced carcinogenesis.

In general, there are two possible post-transcriptional mechanisms accounting for miRNA regulation of its target genes, mRNA cleavage or translational repression, both of which negatively regulate target expression. If an mRNA target is perfectly or near-perfectly complementary to the miRNA, the mRNA will be cleaved and degraded. mRNA translation also can be repressed. Consequently, we identified a number of miRNA targets that show translational repression or mRNA destabilization or both. On the other hand, a number of miRNA targets identified in this study pair with proteins show expression in opposite direction to the change in miRNA expression, but the mRNA level remains unchanged. This is consistent with previous reports that most animal miRNAs complement their targets imperfectly so they function primarily via translational repression rather than mRNA cleavage and degradation [17].

However, we identified a number of miRNA targets with expression of miRNAs and proteins in the same direction. Moreover, we have discovered a network of miRNAs and their functional target interactions associated with AA-induced tumors that show both directions of miRNA - target regulation and most of them are actually expressed in the same direction. In certain cases miRNAs and their potential targets were observed to have similar expression patterns [43-45]. Such a phenomenon might be explained by counter-regulation of different posttranscriptional mechanisms or possibly the down-regulation of protein production being offset by a feedback mechanism [23]. In this study, it remains intriguing that up-regulation of miRNAs such as miR-21 and miR-34a is associated with activation of their target oncoprotein expression after AA treatment for 12 weeks. It remains unknown how miRNAs could maintain the target overexpression that could be a driving force for AA-induced carcinogenesis. So far, miRNA biology studies mainly have focused on miRNA targets with an opposite regulation or a similar expression with the anti-targets. Our research is based on a genome-wide approach and highlights the importance of considering both sides of miRNA regulation, including translational repression resulting in activation in biological functions and diseases.

Finally, we found that there was only a weak correlation between mRNA and protein expression or even opposite expression for a number of genes. One possibility for this lack of association is the different stability of proteins in the rat [46,47]. For example, the half-life of different proteins varies from minutes to days, but the variability for mRNAs is quite a bit less. Also, post-transcriptional and post-translational regulation might affect protein expression. miRNAs are also likely involved in this process and might regulate gene expression through feedback mechanisms, but the mechanisms for these pathways are largely unknown. However, we do not preclude the possibility that mechanisms may be important including transcriptional factors, translational or posttranslational regulation that would affect miRNA target expression.

In summary, we treated rats with a carcinogenic dose of AA and identified dysregulated expression of 63 miRNAs in rat kidney by performing a deep sequencing analysis. IPA analysis indicated that half of them are associated with cancer. We next selected miRNA targets based on those dysregulated miRNAs and combined miRNA data with the RNA microarray and proteomics data in order to understand miRNA regulation of their targets regulation. The ability to compare miRNA, mRNA, and protein expression in the identical samples defined a comprehensive miRNA regulatory network that indicated RNA destabilization, translational repression and activation.

## Conclusions

We employed global profiling of miRNA, transcriptome, and proteome expression in parallel in order to integrate three biological system levels to better understand AA-induced carcinogenesis in rat kidney. AA induced a number of dysregulated miRNAs that are closely associated with cancer initiation and progression. Moreover, the use of integrated approaches helps us better understand the relationships among expression of miRNAs, mRNAs and proteins with a focus on miRNA regulation on genome-wide repression and activation. We identified a cancer-related network that is associated with miRNA target regulation, primarily regulating translational activation. This novel systematic study provides us information on miRNA target regulation on a genome-wide scale. The study supports application of miRNA expression as a biomarker for identifying the genotoxicity and carcinogenicity of AA and other of unknown exposures.

## Methods

### Animal treatment

The treatment schedule was based on the previous carcinogenicity study of AA that resulted in tumors in kidneys and other tissues [27,28]. Specifically, groups of six 6 week-old male Big Blue rats were treated with AA as its sodium salt at 10.0 mg/kg body weight by gavage (4 ml/kg body weight) 5 times a week for 12 weeks, a treatment protocol that induces tumors in rat kidney. Rats treated with 0.9% sodium chloride on the same schedule were used as control. All animals were sacrificed 1 day after the last treatment. The kidneys were isolated, frozen quickly in liquid nitrogen and stored at −80°C. The rats were obtained from Taconic Laboratories (Germantown, NY) and were originally purchased from Stratagene (La Jolla, CA). AA was purchased from Sigma (St. Louis, MO). The purity of AA was 96% and contained 40% of AAI and 56% of AAII. The recommendations set forth by the NCTR Institutional Animal Care and Use Committee were followed for the handling, maintenance, treatment and sacrifice of the rats.

### miRNA isolation

miRNA isolation was performed as previously described [29]. Briefly, 40–50 mg tissue was cut and mechanically minced using Tissue Tearor (Biospec Products Inc, OK). Total RNA was isolated using a small RNA-retaining protocol that employed an organic extraction followed by glass-fiber immobilization (Ambion, TX). RNA concentration was determined with a Nanodrop ND-1000 spectrophotometer (Thermo Scientific, DE). We used sample-specific chips from 2100 Bioanalyzer (Agilent Technologies, Palo Alto, CA) to study RNA quality. The RNA integrity was expressed as RNA integrity number (RIN). We used samples for RNA express array and deep sequencing with high qualities that had a RIN > 8.0.

### Sequencing analysis of miRNA expression

The small RNA library construction and deep sequencing were carried out at the University of Texas Southwestern Medical Center Microarray Core Facility. Samples were prepared using the Illumina Small RNA Sample Prep Kit and following the small RNA v1.5 sample preparation guide. Approximately 10 μg of total RNA was used for small RNA library construction. The v1.5 sRNA 3’ and SRA 5’ adaptors (Illumina, USA) were added to both ends of the small RNA. The 3’ and 5’ ligated RNAs were used as templates for reverse transcription followed by PCR amplification. The enriched cDNA constructs were loaded onto to 6% TBE PAGE gel and the bands containing the 22–30 nt RNA fragments (93–100 nt in length with both adapters) were cut and purified. The concentrations of the libraries were determined using a NanoDrop ND-1000 spectrophotometer and the size and purity were determined using an Agilent 2100 Bioanalyzer in combination with the Agilent DNA 1000 Kit. The purified DNA was used directly for cluster generation and sequence analysis using the Illumina Genome Analyzer II (Illumina, San Diego, CA, USA) according to the manufacturer’s instructions (36 cycle single read cluster kit v4 and sequence kit v4). The image files generated by the sequencer were then processed to produce digital-quality data. After masking of the adaptor sequences and removal of contaminated reads, clean reads were processed for computational analysis. The read numbers for the 4 AA-treated and 4 control animals reached 17.1, 17.1, 16.3, 22, and 16.5, 16.4, 16.7, 19.7 millions, which provided enough coverage depth for miRNA profiling analysis. The miRNA sequencing data was deposited into GEO database with the access number GSE54338.

### Sequencing analysis of mRNA expression

mRNA sequencing was done with an Illumina Genome Analyzer II platform by following the corresponding manufacture protocols. Briefly, poly-A mRNA was isolated from total RNA with Sera-Mag Magnetic Oligo (dT) beads, and fragmented into small pieces using divalent cations under elevated temperature. RNA fragments were then used to synthesize the first and second strand cDNAs using reverse transcriptase and random primers. The ends of cDNA were blunted in an “end repair” reaction with T4 DNA polymerase and Klenow DNA polymerase. An “A” base was then added to the 3’ end of the blunt phosphorylated DNA fragments, which was used to ligate to the Illumina adapter with a single “T” base overhang at its 3’ end. After the ligation reaction, a size range of cDNA templates was selected and amplified on a cluster station with the single-read cluster generation kit v2. Finally, cDNA template clusters were sequenced on an Illumina Genome Analyzer II platform with SBS Sequencing Kit v3. Each RNA sample was sequenced in one lane, generating over 16 million reads of 36 bases long per sample. Base-calling was done by Bustard [48], and sequence analysis was performed with Gerald (http://support.illumina.com/content/dam/illumina-support/documents/myillumina/a557afc4-bf0e-4dad-9e59-9c740dd1e751/casava_userguide_15011196d.pdf). The mRNA sequencing data was deposited into GEO database with the access number GSE21210.

### Mapping of miRNA and mRNA short sequencing reads

We used the miRanalyzer webserver [30] for detection of known miRNAs (http://www.mirbase.org/). The required input in read/count format was generated by means of the provided groupReads.pl perl script. For both analysis steps, conversion of fastq to read/count and analysis of the miRNA expression profiling, we used the default parameters of the programs. To detect differentially expressed genes, we proceeded as following: (i) download all Ensembl transcripts in fasta format (RGSC3.4); (ii) align the reads with Bowtie [31] using a seed alignment of 18 nt and allowing 1 mismatch. Initially we retained the best 10 alignments (−−best --strata) by performing a seed extension to find the best alignment(s) as described in the miRanalyzer paper. Finally we accepted the alignments if equal or less than the 9 best alignments (after the seed extension) existed. (iii) We calculated the read count for each reference sequence (Ensembl transcript). Note that by doing so, a read with multiple mappings will contribute to the total read count of several transcripts. Another way would be to distribute the read count equally over the multiple mapped transcripts (dividing the read count of an ambiguously mapped read by the number of mappings). However, this would lead to non-integer read counts which might cause inappropriate mismatches between mRNAs and microRNAs in DESeq. Moreover, we are interested in differentially expressed mRNA and therefore the effect of the multiple mapping treatments will cancel out between the two conditions.

### Data normalization and selection of differentially expressed miRNAs (DEMs) and differentially expressed genes (DEGs)

We used the DESeq bioconductor package to detect differentially expressed miRNAs and mRNAs [31]. miRanalyzer incorporates a differential expression module based on DESeq. For the mRNA sequencing data, we built an expression matrix with raw read counts of all transcripts and conditions that are input for DESeq.

### Classification of miRNA and mRNA expression profile

All of the detectable miRNAs were applied to PCA [49] and HCA [50] to determine the global effects of AA treatment on miRNA expression. The analysis was done within ArrayTrack™, an FDA-developed software package for managing, analyzing, and interpreting microarray gene expression data [51]. The sequencing data were normalized to let each sample have the same number of reads of 1 million, log2 transformed, and scaled (letting all the genes have the mean of 0 and standard deviation of 1 across the 8 samples) prior to analysis. PCA were conducted using the autoscaled method. For HCA, the distance matrix was calculated using the Euclidean method and the dendrogram was linked with average algorithm.

### Functional analysis of the DEMs

The functional relevance of the DEMs was analyzed within IPA systems (http://www.ingenuity.com/), an online functional analysis software that annotates the genes in terms of biological functions. Also, we used IPA to pull out cancer related miRNAs and cancer-related miRNA targets from miRTarBase and link them together. Fisher’s exact test was used to evaluate the function changes. A function with a p-value < 0.05 was considered as significantly affected by the AA treatment.

### Proteomic analysis of protein changes in rat kidneys

The proteomic analysis of the same set of rat kidneys for miRNA/mRNA profiling was performed at Center for Proteomics, National Center for Toxicological Research (NCTR) by following a standard procedure as detailed in a previous study [52]. Briefly, proteomes of rat kidney were quantitatively analyzed using trypsin catalyzed ^16^O/^18^O labeling in conjunction with two-dimensional liquid chromatography separation and tandem mass spectrometry (2DLC-MS/MS). More than 9000 unique peptide sequences, were identified and quantified. The false discovery rate (FDR) was estimated to be < 2% at the peptide level for these peptide datasets as a result of a reversed protein sequence database search. Evaluation of quantification errors and statistic analysis was performed for the identification of reliable protein expression changes as a result of AA treatment. p < 0.05 to define differentially expressed proteins.

## Additional files

Additional file 1: Table S1. The differentially expressed microRNAs in AA-treated rat kidney. Additional file 2: Figure S1. TaqMan confirmation of 6 microRNA expressions determined by NGS. Six microRNAs, which have low, middle, or high expression levels, significant or non-significant expression changes, and down or up change directions, were selected to do TaqMan real-time PCR. Shown here are the fold changes detected by two technologies. Additional file 3: Figure S2. IPA analysis of differential mRNAs treated with AA in rat kidney. (A) The canonical pathway of 4051 upregulated genes. (B) The canonical pathway of 2743 downregulated genes. Additional file 4: Figure S3. Correlation among miRNAs, mRNAs, and proteins. We conducted genome-wide correlation analyses of expression changes between mRNAs vs. protein, miRNA vs. mRNA, and miRNA vs. protein in kidney in rats treated with AA. The Graphpad Prism 5.1 was used for this analysis. We calculated the correlation coefficient R and P value. P < 0.05 indicates significant difference.

## Abbreviations

AA: Aristolochic Acid
microRNA: miRNA
DEM: Differentially expressed miRNA
DEG: Differentially expressed genes
PCA: Principal component analysis
HCA: Hierarchical clustering analysis
IPA: Ingenuity Pathway Analysis
