# Supplementary material for: Integrated microRNA, mRNA, and protein expression profiling reveals microRNA regulatory networks in rat kidney treated with a carcinogenic dose of aristolochic acid
Source: BMC Genomics. 2015 May 8;16(1):365. doi: 10.1186/s12864-015-1516-2 (PMC4456708; doi:10.1186/s12864-015-1516-2)
Supplement: Additional file 1: Table S1. — The differentially expressed microRNAs in AA-treated rat kidney. [file 12864_2015_1516_MOESM1_ESM.doc]

**Supplementary Table 1. The differentially expressed microRNAs in AA-treated rat kidney**

| **microRNA** | **Mean_CTL** | **Mean_AA** | **Fold Change** | **Adjusted p value** |
| --- | --- | --- | --- | --- |
| rno-miR-881 | 0.6 | 34.4 | 56.28 | 0 |
| rno-miR-880 | 0.3 | 9.4 | 34.37 | 0.000037 |
| rno-miR-741-3p | 4.1 | 99.8 | 24.28 | 0 |
| rno-miR-34a | 384.8 | 8250.7 | 21.44 | 0 |
| rno-miR-511* | 199.7 | 3967.6 | 19.86 | 0 |
| rno-miR-34a* | 2.3 | 37.8 | 16.76 | 0 |
| rno-miR-34b | 8.5 | 116.2 | 13.59 | 0 |
| rno-miR-34c | 5120.4 | 68642.3 | 13.41 | 0 |
| rno-miR-449c-5p | 0.5 | 6.1 | 12.79 | 0.005178 |
| rno-miR-187 | 102.2 | 1193 | 11.67 | 0 |
| rno-miR-34c* | 21.3 | 233.3 | 10.96 | 0 |
| rno-miR-449a | 9.3 | 98.3 | 10.57 | 0 |
| rno-miR-34b* | 25.9 | 261.3 | 10.1 | 0 |
| rno-mir-146b* | 1.1 | 10.7 | 9.9 | 0.000184 |
| rno-miR-124 | 1.5 | 13.4 | 9.23 | 0.000037 |
| rno-miR-224 | 23.6 | 209.4 | 8.89 | 0 |
| rno-miR-122 | 239.7 | 1896.9 | 7.91 | 0 |
| rno-miR-3085 | 1 | 7.6 | 7.26 | 0.004674 |
| rno-miR-122* | 8.9 | 58.9 | 6.64 | 0 |
| rno-miR-187* | 2.2 | 14.2 | 6.56 | 0.000316 |
| rno-miR-130b* | 1.6 | 9.7 | 6.18 | 0.003434 |
| rno-miR-205 | 331.3 | 1983.4 | 5.99 | 0 |
| rno-miR-224* | 1.4 | 7 | 5.13 | 0.02173 |
| rno-miR-130b | 130.4 | 620.2 | 4.75 | 0 |
| rno-miR-466c | 5.3 | 24.9 | 4.69 | 0.000433 |
| rno-miR-146b | 5619 | 26348.1 | 4.69 | 0 |
| rno-miR-132 | 135.6 | 525.6 | 3.88 | 0.000008 |
| rno-miR-21 | 476470.1 | 1825993 | 3.83 | 0 |
| rno-miR-301b | 12 | 44.7 | 3.71 | 0.001053 |
| rno-miR-31* | 54.4 | 197.1 | 3.62 | 0.000133 |
| rno-miR-212 | 24.6 | 86 | 3.5 | 0.0013 |
| rno-miR-466b-2* | 16.5 | 57.3 | 3.47 | 0.001976 |
| rno-miR-21* | 407.8 | 1223 | 3 | 0.000072 |
| rno-miR-503 | 1293.6 | 3492.4 | 2.7 | 0.000038 |
| rno-miR-142-3p | 14046.6 | 37532.2 | 2.67 | 0 |
| rno-miR-708 | 481.2 | 1243.8 | 2.58 | 0.00068 |
| rno-miR-18a | 294.6 | 659.1 | 2.24 | 0.020082 |
| rno-miR-708* | 166.8 | 365.8 | 2.19 | 0.029964 |
| rno-miR-450a | 1659.2 | 3301.1 | 1.99 | 0.008409 |
| rno-miR-223 | 590.1 | 1146.6 | 1.94 | 0.038581 |
| rno-miR-31 | 632 | 1185.8 | 1.88 | 0.037226 |
| rno-miR-542-3p | 10320.4 | 19224.6 | 1.86 | 0.004674 |
| rno-miR-322 | 3279.1 | 5887.1 | 1.8 | 0.030794 |
| rno-miR-191 | 122511.2 | 76337 | -1.6 | 0.029964 |
| rno-miR-140* | 209371.7 | 126391.1 | -1.66 | 0.017962 |
| rno-miR-194 | 32436 | 19030.4 | -1.7 | 0.026503 |
| rno-miR-148b-3p | 101605.2 | 58938.8 | -1.72 | 0.013215 |
| rno-miR-378 | 4260974 | 2459046 | -1.73 | 0.002941 |
| rno-miR-152 | 69300.4 | 39634 | -1.75 | 0.014206 |
| rno-miR-192 | 567860.1 | 316369.5 | -1.79 | 0.007764 |
| rno-miR-653 | 1079.2 | 471.9 | -2.29 | 0.019794 |
| rno-miR-103 | 247338.9 | 106639.4 | -2.32 | 0.000007 |
| rno-miR-3545-3p | 16861.6 | 6822.7 | -2.47 | 0.000168 |
| rno-miR-494 | 51.1 | 19.8 | -2.58 | 0.044158 |
| rno-miR-135a | 1060.6 | 385 | -2.75 | 0.001397 |
| rno-miR-135b | 103.6 | 37.6 | -2.76 | 0.017213 |
| rno-let-7e* | 56.8 | 19.4 | -2.92 | 0.00251 |
| rno-miR-382* | 56.3 | 18.5 | -3.04 | 0.010564 |
| rno-miR-129-2* | 49.7 | 13.8 | -3.6 | 0.00251 |
| rno-miR-135a* | 20.4 | 4.6 | -4.43 | 0.005406 |
| rno-miR-375 | 3652.8 | 642.5 | -5.69 | 0 |
| rno-miR-219-2-3p | 131.6 | 17 | -7.72 | 0 |
| rno-miR-383 | 18.2 | 1 | -18.14 | 0 |

Note:1) Mean_CTL and Mean_AA refer to the mean value of DESeq-normalized read counts for 4 control and 4 AA-treated samples. Fold change is equal to Mean_AA / Mean_CTL when Mean_AA is larger than Mean_CTL or -(Mean_CTL/Mean_AA) when Mean_AA is less than Mean_CTL. 2) Among the 63 DEM, 31 was cancer related. These includes rno-miR-708, rno-miR-542-3p, rno-miR-511, rno-miR-503, rno-miR-494, rno-miR-450a, rno-miR-383, rno-miR-375, rno-miR-34a, rno-miR-31, rno-miR-224, rno-miR-223, rno-miR-21, rno-miR-205, rno-miR-194, rno-miR-192, rno-miR-191,rno-miR-18a, rno-miR-187, rno-miR-148b-3p, rno-miR-146b, rno-miR-142-3p, rno-miR-140*,rno-miR-135a, rno-miR-132, rno-miR-130b, rno-miR-129-2*, rno-miR-124, rno-miR-122, rno-miR-103, rno-let-7e*.
