# Supplementary material for: Integrated microRNA, mRNA, and protein expression profiling reveals microRNA regulatory networks in rat kidney treated with a carcinogenic dose of aristolochic acid
Source: BMC Genomics. 2015 May 8;16(1):365. doi: 10.1186/s12864-015-1516-2 (PMC4456708; doi:10.1186/s12864-015-1516-2)
Supplement: Additional file 2: Figure S1. — TaqMan confirmation of 6 microRNA expressions determined by NGS. Six microRNAs, which have low, middle, or high expression levels, significant or non-significant expression changes, and down or up change directions, were selected to do TaqMan real-time PCR. Shown here are the fold changes detected by two technologies. [file 12864_2015_1516_MOESM2_ESM.doc]

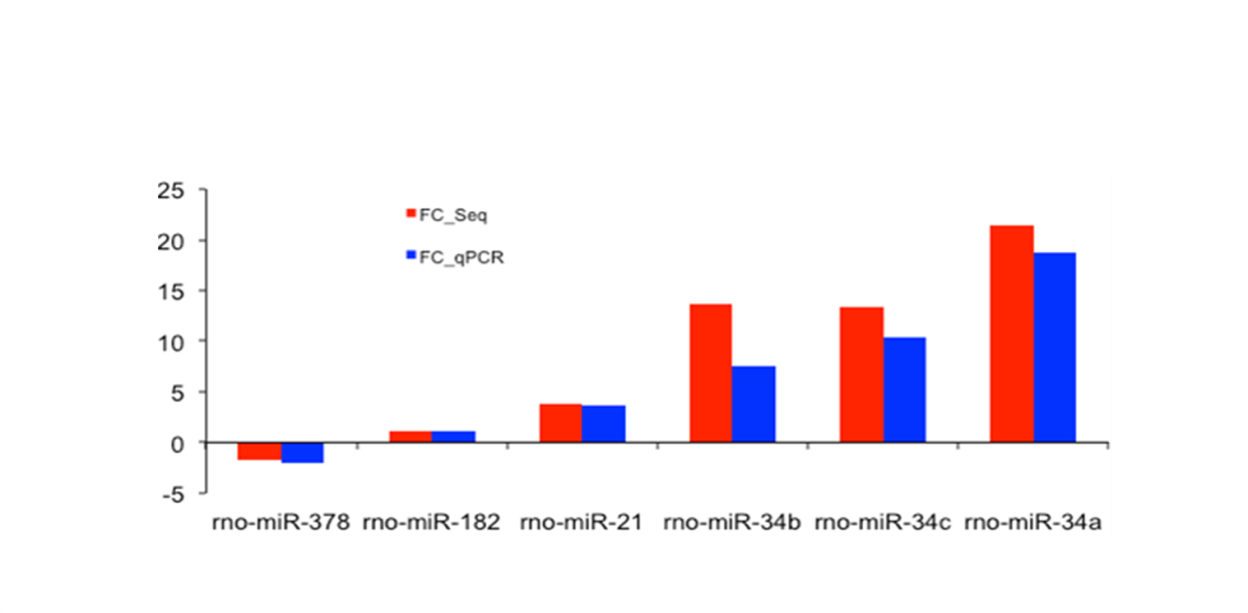


Supplementary Figure1. TaqMan confirmation of 6 microRNA expressions determined by NGS. Six microRNAs, which have low, middle, or high expression levels, significant or non-significant expression changes, and down or up change directions, were selected to do TaqMan real-time PCR. Shown here are the fold changes detected by two technologies. (From the file “Sig miRNAs_qPCR confirmation_20110302.xls “)
