# Supplementary material for: Integrated microRNA, mRNA, and protein expression profiling reveals microRNA regulatory networks in rat kidney treated with a carcinogenic dose of aristolochic acid
Source: BMC Genomics. 2015 May 8;16(1):365. doi: 10.1186/s12864-015-1516-2 (PMC4456708; doi:10.1186/s12864-015-1516-2)
Supplement: Additional file 3: Figure S2. — IPA analysis of differential mRNAs treated with AA in rat kidney. (A) The canonical pathway of 4051 upregulated genes. (B) The canonical pathway of 2743 downregulated genes. [file 12864_2015_1516_MOESM3_ESM.doc]

A


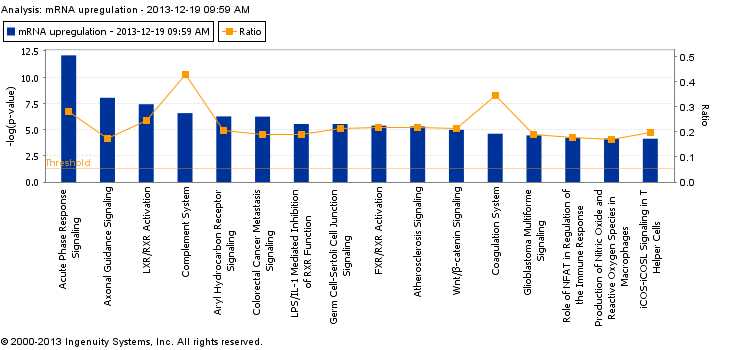


B


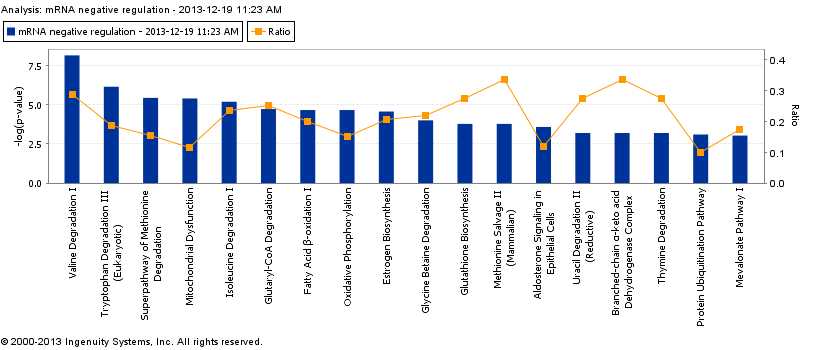


Supplementary Figure 2. IPA analysis of differential mRNAs treated with AA in rat kidney. (A) The canonical pathway of 4051 upregulated genes. (B) The canonical pathway of 2743 downregulated genes.
