# Supplementary material for: Integrated microRNA, mRNA, and protein expression profiling reveals microRNA regulatory networks in rat kidney treated with a carcinogenic dose of aristolochic acid
Source: BMC Genomics. 2015 May 8;16(1):365. doi: 10.1186/s12864-015-1516-2 (PMC4456708; doi:10.1186/s12864-015-1516-2)
Supplement: Additional file 4: Figure S3. — Correlation among miRNAs, mRNAs, and proteins. We conducted genome-wide correlation analyses of expression changes between mRNAs vs. protein, miRNA vs. mRNA, and miRNA vs. protein in kidney in rats treated with AA. The Graphpad Prism 5.1 was used for this analysis. We calculated the correlation coefficient R and P value. P < 0.05 indicates significant difference. [file 12864_2015_1516_MOESM4_ESM.doc]

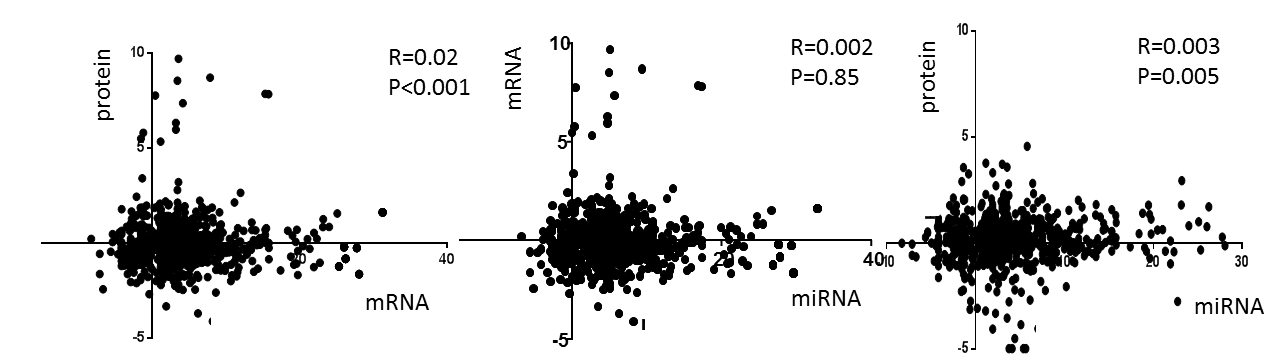


Supplementary Figure 3. Correlation among miRNAs, mRNAs, and proteins. We did genome-wide correlation analyses of expression changes between mRNAs vs. protein, miRNA vs. mRNA, and miRNA vs. protein in kidney in rats treated with AA. The Graphpad Prism 5.1 was used for this analysis. We calculated the correlation coefficient R and P value. P<0.05 indicates significant difference.
